# Variable number of TMC1-dependent mechanotransducer channels underlie tonotopic conductance gradients in the cochlea

**DOI:** 10.1038/s41467-018-04589-8

**Published:** 2018-06-05

**Authors:** Maryline Beurg, Runjia Cui, Adam C. Goldring, Seham Ebrahim, Robert Fettiplace, Bechara Kachar

**Affiliations:** 1 Department of Neuroscience, University of Wisconsin School of Medicine and Public Health, Madison, WI 53706 USA; 2 Laboratory of Cell Structure and Dynamics, National Institute on Deafness and Other Communication Disorders, National Institutes of Health, Bethesda, MD 20892 USA; 3 Laboratory of Cellular and Molecular Biology, Center for Cancer Research, National Cancer Institute, National Institutes of Health, Bethesda, MD 20892 USA

## Abstract

Functional mechanoelectrical transduction (MET) channels of cochlear hair cells require the presence of transmembrane channel-like protein isoforms TMC1 or TMC2. We show that TMCs are required for normal stereociliary bundle development and distinctively influence channel properties. TMC1-dependent channels have larger single-channel conductance and in outer hair cells (OHCs) support a tonotopic apex-to-base conductance gradient. Each MET channel complex exhibits multiple conductance states in ~50 pS increments, basal MET channels having more large-conductance levels. Using mice expressing fluorescently tagged TMCs, we show a three-fold increase in number of TMC1 molecules per stereocilium tip from cochlear apex to base, mirroring the channel conductance gradient in OHCs. Single-molecule photobleaching indicates the number of TMC1 molecules per MET complex changes from ~8 at the apex to ~20 at base. The results suggest there are varying numbers of channels per MET complex, each requiring multiple TMC1 molecules, and together operating in a coordinated or cooperative manner.

## Abstract (web summary)

Mechanoelectrical transduction channel (MET) current found in stereocilia of hair cells matures over the first postnatal week. Here the authors look at the contribution of transmembrane channel-like protein 1 and 2 (TMC1 and TMC2) to MET current during development of tonotopic gradients.

## Introduction

Cochlear hair cells detect sound stimuli via opening of mechanoelectrical transduction (MET) channels in the sensory hair bundle. In mice, MET develops during the first postnatal week, over which period there are modifications in the organization of the hair bundle, including growth of the component stereocilia and reabsorption of supernumerary microvilli^1,2^. There are also changes in the biophysical properties of the MET channels, with the magnitude of the MET current and the extent and speed of adaptation increasing to reach mature levels^3–5^. As with other aspects of cochlear development^6^, maturation of high-frequency hair cells at the base of the cochlea precedes apical low-frequency hair cells by about 2 days. Why the MET current is needed prior to the onset of hearing, and what mechanisms underlie the changes in properties are unknown.

The MET channels are located at the tips of all but the tallest stereocilia^7^, and they are thought to be activated by tension in extracellular tip links^7–9^. The MET channels have large unitary conductance increasing several-fold from apex to base in OHCs^9,10^. Whether the gradient in conductance is continuous, and what is its mechanistic origin are unknown, as is the molecular composition of the MET channels. However, there is evidence for a central role of transmembrane channel-like protein isoforms 1 and 2 (TMC1 and TMC2)^11,12^, as well as other components such as LHFPL5^13^ and TMIE^14^. The two TMC isoforms have been localized to the MET channel site at the tips of the shorter stereocilia in mature hair cells^15^. Mutations in either isoform alter ion conduction through the MET channels^5,16,17^. TMC1 null mice are deaf and lack MET currents in OHCs after postnatal day (P)8^5,12^ prior to the onset of hearing at around P12^18^. However, in *Tmc1* mutants, MET currents occur before P8, when they are supported by TMC2, which can initially substitute for TMC1 but ultimately disappears after the first week^12,15^. It has been reported that TMC2 cannot rescue hearing in adult mice lacking TMC1^12,15^.

Several reports suggest that MET function controls, (via tip link tension or Ca^2+^ homeostasis), stereociliary properties^19–21^. Since the molecular machineries associated with the MET channel and with regulation of the stereociliary length^22–24^ co-exist within the MET channel site at the stereociliary tip^7^, it is unsurprising that the two processes are linked^19,25,26^. While it has been reported that the absence of TMC1 ultimately leads to bundle degeneration^12,27^, what role TMC1 and TMC2 play in the development of the stereociliary bundle is unknown. We examine the relative contributions of TMC1 and TMC2 to MET channel conductance during development, and address the changes in this property along the cochlea’s tonotopic axis^4,9,28^. We show that TMCs are differentially required for development of the tonotopic gradients in stereociliary bundle structure and MET channel properties. This influence correlates with increasing numbers of TMC molecules per putative MET site from the cochlear apex to base.

## Results

### Contributions of TMC1 and TMC2 to MET current development

**Fig. 1:**
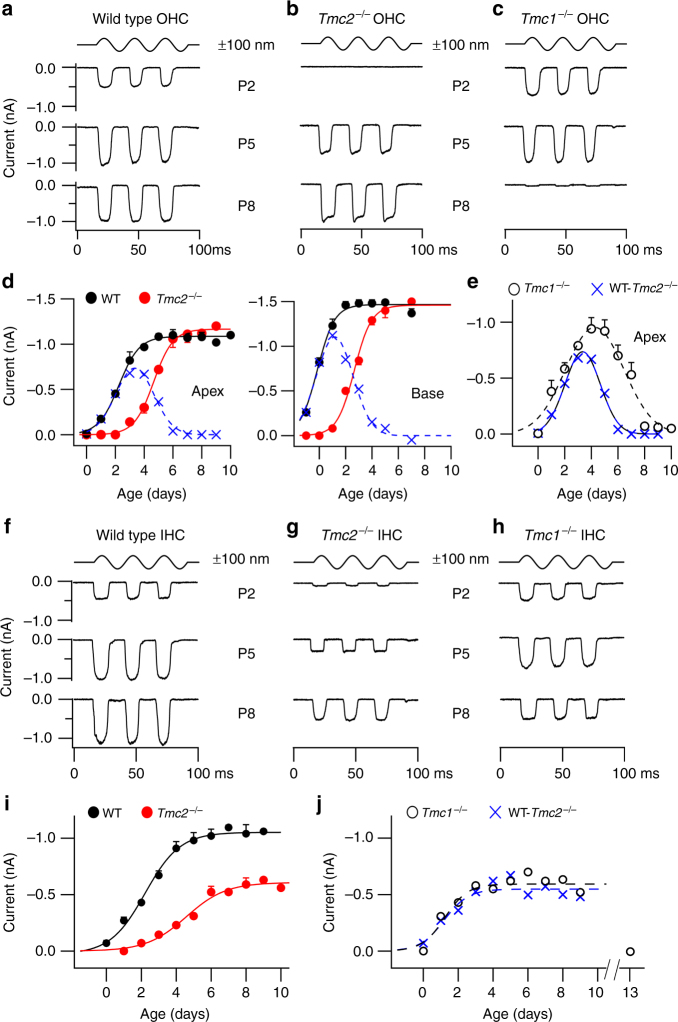
Contribution of TMC2 to development of mechanotransduction in OHCs and IHCs. **a** Examples of maximum MET currents of wild-type apical OHCs during the first postnatal week, ages P2, P5, and P8. Top trace denotes timing of 100 nm amplitude sinusoidal vibration of the hair bundle evoked by a fluid jet stimulator. **b** MET currents in *Tmc2*^−/−^ in apical OHCs. **c** MET currents in *Tmc1*^−/−^ mouse apical OHCs. **d** MET current amplitudes (mean ± SEM) in wild type (WT; black symbols) and *Tmc2*^−/−^ (red symbols) in OHCs from cochlear apex and base. Numbers of OHCs for points with increasing age: apex WT, 13, 6, 5, 8, 6, 6, 5; apex *Tmc2*^*−/−*^, 3, 3, 3, 5,7, 5, 6, 3, 3; apex *Tmc1*^*−/−*^: 3, 8, 5, 4, 7, 9, 3, 6, 8, 6, 3; base WT: 7, 7, 4, 7, 9, 12, 7, 3; base *Tmc2*^*−/−*^: 3, 3, 4, 5, 5, 4, 4, 3. **e** Expression of TMC2 in apical OHCs, derived as the difference between wild type and *Tmc2*^−/−^ (crosses) and from *Tmc1*^−/−^ mice (open symbols); points fit with Gaussian curves. Numbers of cells: WT, 5 to 13; *Tmc2*^*−/−*^ mice 3 to 7. *Tmc1*^*−/−*^ mice 5 to 7. **f** Examples of maximum MET currents during development of wild type apical IHCs at postnatal ages P2, P5 and P8. **g** MET currents during same period in apical IHCs from *Tmc2*^−/−^ mice. **h** MET currents in apical IHCs from *Tmc1*^*−/−*^ mice at postnatal ages P2, P5, and P8. **i** MET currents (mean ± SEM) in wild type (black symbols) and *Tmc2*^*−/−*^ (red symbols). **j** Expression of TMC2 in IHCs, derived as the difference between wild type and *Tmc2*^−/−^ (blue crosses, fit with blue dashed line) and from *Tmc1*^−/−^ mice (open symbols, fit with black dashed line). Numbers of cells, increasing in age: IHC, WT: 5, 10, 6, 10, 6, 6, 5, 4, 4, 4; IHC *Tmc2*^*−/−*^ 3, 9, 11, 8, 6, 7, 6, 5, 3, 3; IHC *Tmc1*^*−/−*^ 3, 4, 5, 3, 4, 5, 4, 4, 4, 4, 3. Holding potential −84 mV

In *Tmc2*^*−/−*^ mice the MET current in OHCs increased in size with development over 3 days to reach the same peak amplitude as in the wild type, but its half maximum was delayed ~2.6 days relative to wild type (Fig. 1a–d). The amplitude of the OHC MET current was half-maximal in the wild type at 2.2 ± 0.06 days (apex) and −0.2 ± 0.04 days (base) and in *Tmc2*^*−/−*^ mice was 4.6 ± 0.06 days (apex) and 2.6 ± 0.1 days (base). If TMC1 and TMC2 are the only determinant elements of the MET channel current, then the results with *Tmc2*^*−/−*^ mice can be used to establish the contribution of TMC1. At a given age, the difference in current between the wild type and *Tmc2*^*−/−*^ should therefore yield the TMC2-dependent currents, which in OHCs is a bell-shaped function (Fig. 1e, crosses). Expression of TMC2 was also derived from recordings in *Tmc1*^*−/−*^ mice (Fig. 1e, open symbols). This method also gave a bell-shaped function, but it peaked later with a greater amplitude than found with the subtraction method (Fig. 1e). The non-additive influence suggests that absence of TMC1 upregulates or prolongs expression of TMC2.

The same analysis was applied to apical IHCs (Fig. 1f–j), for which the MET current increased over 4–5 days to reach 1.05 ± 0.02 nA, with a half-maximal time, *d*_0.5_, at 2.5 ± 0.1 days. In *Tmc2*^−/−^ mice the growth in MET current was delayed by 2 days (*d*_0.5_ = 4.5 ± 0.3 days). Unlike OHCs, the plateau current in the *Tmc2*^*−/−*^ animals was only half that of the wild type. Consistent with this result, the current in *Tmc1*^*−/−*^ mice did not become zero over the time period studied. At P9, the MET current in *Tmc1*^*−/−*^ mice was 0.44 ± 0.03 nA (*N* = 3). However, by P13, at the onset of hearing, in the *Tmc1*^*−/−*^ mice, no current was measurable (*N* = 3), suggesting that TMC2 was absent by this stage. In the wild type, a significant MET current of 0.42 ± 0.03 nA (*N* = 3) was still recordable at P13 though smaller than at P8. Such a reduction may partly stem from damage to the bundle, but could also reflect a real loss of channels. The development of the MET current in IHCs differs from that in OHCs in that it takes 50% longer (4.4 days compared to 3.1 days), and TMC2 persists later than in OHCs.

### Single MET channel records show multiple conductance levels

**Fig. 2:**
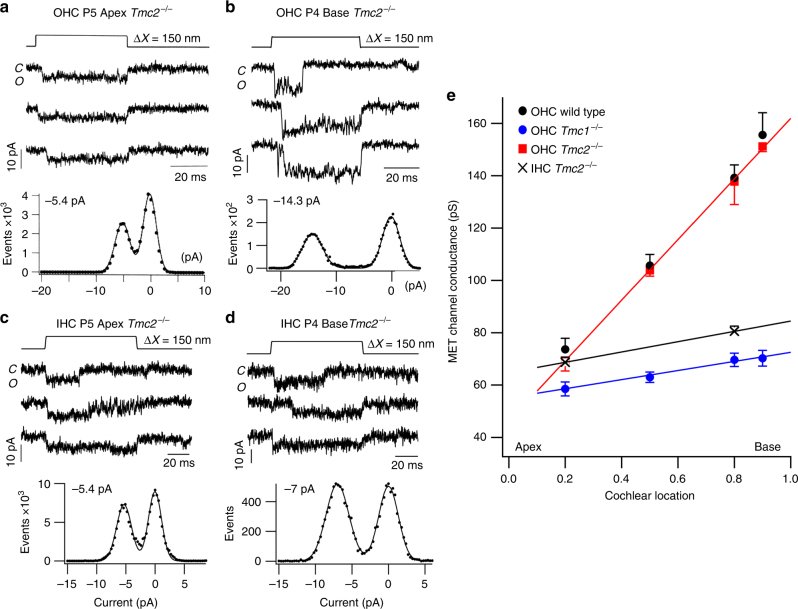
Tonotopic organization of MET channels. **a** Three examples of single-channel currents in response to hair bundle deflection (top) in P5 apical *Tmc2*^*−/−*^ mice. Amplitude histogram of events gives −5.4 pA single-channel current. **b** Three examples of single-channel currents in P4 basal OHC; amplitude histogram of events gives −14.3 pA single-channel current. Holding potential −84 mV. **c** Three examples of single-channel currents evoked in apical IHC of P5 *Tmc2*^*−/−*^ mice. Amplitude histogram of events gives 5.4 pA single-channel current. **d** Three examples of single-channel currents in P4 basal IHC; amplitude histogram of events gives 6.9 pA single-channel current. Holding potential −84 mV. **e** Collected results on MET channel conductance plotted against fractional distance along the cochlea from the apex, for OHCs of P3–P7 wild-type (black symbols), *Tmc2*^*−/−*^ mice (red symbols), and *Tmc1*^*−/−*^ mice (blue symbols) and for *Tmc2*^*−/−*^ mouse IHCs (black crosses). Each point is the mean ± SEM; numbers of cells, apex to base: OHC wild type, 14, 4,14, 7; OHC *Tmc2*^*−/−*^ 11, 4, 10, 3; OHC *Tmc1*^*−/−*^18, 3, 14, 3; IHC *Tmc2*^*−/−*^ 7, 5. Lines drawn through points for *Tmc2*^*−/−*^ and *Tmc1*^*−/−*^ mice. MET channels for *Tmc2*^*−/−*^ mice still exhibit a tonotopic conductance gradient like wild type, but the gradient is virtually absent in *Tmc1*^*−/−*^ mice indicating the tonotopic conductance gradient is supported by TMC1 but not TMC2. All measurements in 1.5 mM extracellular Ca^2+^

**Fig. 3:**
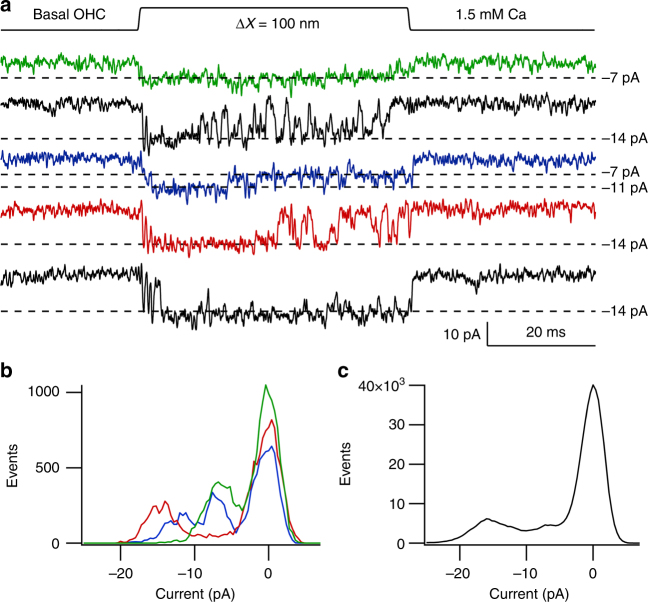
Multiple single-channel levels in a basal OHC. **a** Five exemplar traces showing channel events during 100 nm bundle deflection. The principal current levels are indicated by dashed lines corresponding to −7, −11, and −14 pA. **b** Amplitude histograms of the three colored traces showing the three levels of −7, −11 and −14 pA. **c** Amplitude histogram of all 41 traces from this recording. The levels are smeared so it is difficult to infer specific levels other than the largest one. Note the smearing was not always evident (Fig. 2b). OHC from P4 Tmc2^−/−^ mouse; 1.5 mM external Ca^2+^; holding potential −84 mV

Measurements of single MET channels have shown a tonotopic gradient in unitary conductance in wild-type OHCs, and this gradient is largely absent in the *Tmc1*^*−/−*^ mice^9,29^. However, the gradient persists in *Tmc2*^*−/−*^ mice. Thus, when only TMC1 is present (in *Tmc2*^*−/−*^ mice), the gradient in channel conductance is virtually identical to that in wild type, but when only TMC2 is present (in *Tmc1*^*−/−*^ mice) the gradient is significantly reduced (Fig. 2). The ratio of MET conductance at 90% of the distance along the cochlea from the apex relative to that at 20% was 2.2 with only TMC1 compared to 1.2 with only TMC2. A similarly small apex–base gradient of 1.2 was seen in IHCs (Fig. 2), consistent with the lack of significant gradient in the macroscopic MET current^5,30^. In the previous analysis of single MET-channels, a principal channel current level was inferred from the amplitude histograms of the sweeps. However, there were suggestions in some traces of multiple levels of channel opening, exemplified by the channel records in some high-frequency OHCs (Fig. 3). Amplitude histograms of three individual traces showed a closed level and one or more open levels (Fig. 3b). Applying this procedure to all available traces (41) gave current levels of −7.1 ± 0.4 pA (*N* = 20), −11.2 ± 0.8 pA (*N* = 18) and −14.5 ± 0.9 pA (*N* = 27). Nevertheless, if a single-amplitude histogram was constructed from all 41 traces, the levels were smeared out and it was impossible to extract individual levels (Fig. 3c).

**Fig. 4:**
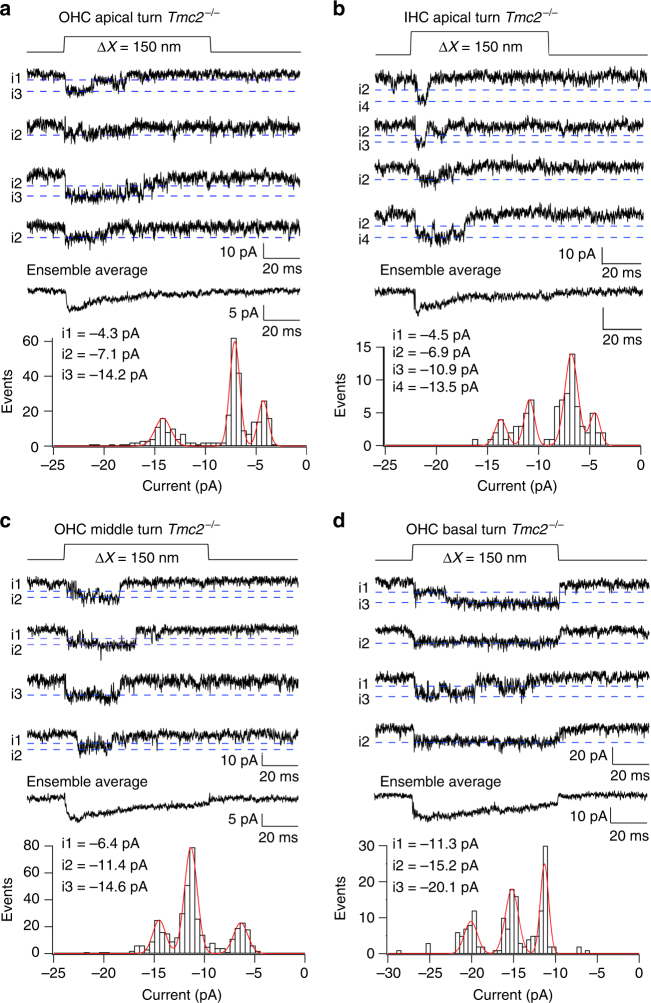
MET single-channel distributions in OHCs and IHCs in *Tmc2*^*−/−*^ mice. **a** Four examples of single-channel current events in a P4 apical OHC, and ensemble average of 100 presentations of this bundle stimulus. Amplitude histogram of current levels, shown below fitted with Gaussian of current levels i1, i2, and i3 denoted by blue dashed lines superimposed on single-channel traces. Current levels and standard deviation of Gaussian fits: −4.3, 0.5 pA; −7.1, 0.5 pA; −14., 0.7 pA. **b** Examples of single-channel current events in a P6 apical IHC, and ensemble average of 100 presentations of this bundle stimulus. Current levels and standard deviation: −4.5, 0.6 pA, −6.9, 0.7 pA; −10.9, 0.64 pA; −13.5, 0.64 pA. **c** Four examples of single-channel current events in P3 middle turn OHC, and ensemble average of 100 stimulus presentations. Current levels and standard deviation: −6.4, 0.7 pA; −11.4, 0.6 pA; −14.6, 0.6 pA.** d** Four examples of single-channel current events in P3 basal OHC and ensemble average of 100 presentations of this bundle stimulus at top. Current levels and standard deviation: ; −11.3, 0.5 pA; −15.2, 0.57 pA; −20.1, 0.7 pA. Holding potential −84 mV in all experiments. All measurements in low (0.04 mM) extracellular Ca^2+^

The possibility of multiple levels was further investigated by recording channel activity after tip-link disruption, but bathing the hair bundle in reduced (0.04 mM) extracellular Ca^2+^ to augment channel amplitude^31^ thereby improving signal to noise ratio. Two approaches were used to infer current levels. In one, current records were analyzed by manual inspection of each trace for discrete current levels (see Methods; Supplementary Fig. 1), quantifying each level after zeroing a short sequence prior to the opening transition; in the other, amplitude histograms of individual traces were constructed as in Fig. 3. In applying the former method, the tonotopic variation in TMC1-containing channels was studied by comparing MET channel events in a total of ten *Tmc2*^*−/−*^ OHCs, three apical, three middle and four basal cochlear regions. Transitions between different levels were evident in most recordings (Fig. 4), with each cell showing at least two or more current levels. Current amplitudes in each of the sweeps were compiled into a histogram and fit with a sum of Gaussians (Fig. 4). The dominant levels, inferred from the peaks of the Gaussians, were similar across cells, with current values (mean ± SD; *N* = number of cells), at −84 mV holding potential, of −4.2 ± 0.3 pA (*N* = 3), −7.1 ± 0.8 pA (*N* = 8), −11.3 ± 0.6 pA (*N* = 6), −14.7 ± 1.3 pA (*N* = 8), and −20.5 ± 1.4 pA (*N* = 3). The largest current of −20.5 pA was present only in basal OHCs and the smallest current level of − 4.2 pA was only seen in apical cells (Fig. 4a, b).

**Fig. 5:**
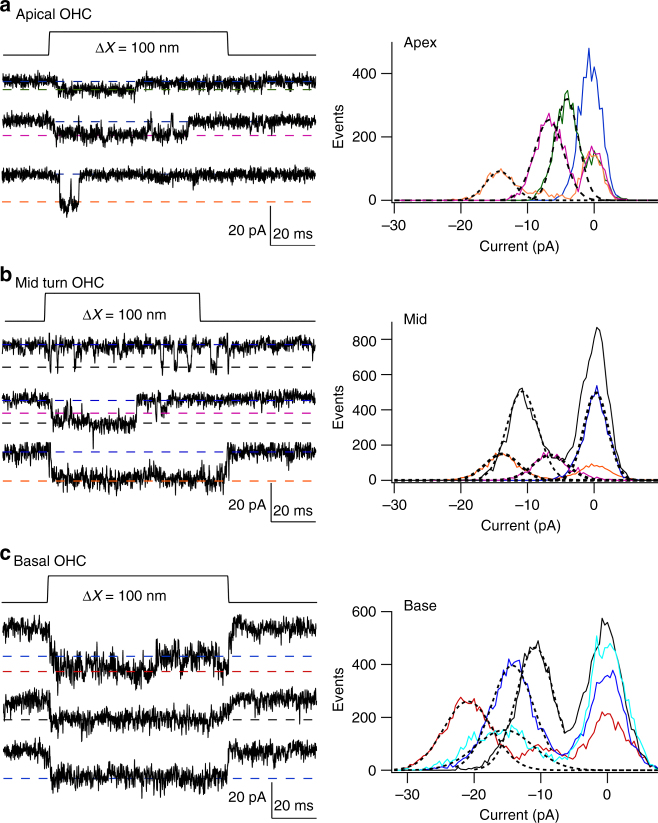
Histograms of multiple-level channel events. **a** Apical OHC, three traces of channel responses to 100 nm step deflection of the hair bundle, with peak levels (corresponding to *x*o values) drawn as dashed lines. Histograms of amplitude distributions of segments of these and three other traces, with different levels, fit with Gaussians = *A* exp{−(*x*−*x*o)^2^/2*σ*^2^} (black dotted lines superimposed on noisy data) with values of *x*o and *σ*: −4.1, 1.7 pA; −6.7, 2.2 pA; −14.2, 2.1 pA. **b** Middle turn OHC, three traces. Gaussian fits with values of *x*o and σ: −6.6, 3.7 pA; −10.8, 3.3 pA; −13.8, 3.6 pA. For the brief openings, exemplified in top trace, five histograms were summed to produce the green curve. **c** Basal turn OHC, three traces, and Gaussian fits with values of *x*o and *σ*: −10.8, 2.8 pA; −14.2, 3.6 pA; −15.4, 4.2 pA; −20.9, 3.6 pA. holding potential −84 mV. For all locations, each amplitude histogram (color) represents the sum of multiple analyses. The analyses in (**b**) and (**c**) are from the same pool of data as in Fig. 4c, d

The existence of multiple conductance states had gone unrecognized because they were not prominent in the usual amplitude histograms (Fig. 2), at least partly due to masking by steady current noise. However, after finding that more than one current level was present as revealed by the method employed above, sections of records were subsequently analyzed by constructing conventional histograms in some cells with low background noise (Fig. 5) from single traces with longer openings. These showed that different traces displayed different peaks in the amplitude histogram distinct from the closed state. Each level was fit with a Gaussian with peak current comparable to those deduced from the manual inspection. Figure 5 shows only three overlapping histograms for each cell, but more complete results were obtained by applying this method to all sweeps with channels in these cells (Table 1). The manual method (Fig. 4) and histogram method (Fig. 5) yielded similar results; for example, the mean values from the manual method in the basal OHC (Fig. 4d) were −11.2, −15.2, and −20.1 pA, whereas the values from the histogram method (Table 1) were −6.2, −11.3, −14.7, and −20.7 pA. This analysis also confirmed that there is an overlap in current levels between apical and basal OHCs, but with different relative representations (Table 1). It should be noted that the MET currents in 0.04 mM Ca^2+^ are 40–50% larger than in 1.5 mM Ca^2+^ ^9^. Converting the amplitudes from the basal OHC in Fig. 3 to those in 0.04 mM Ca^2+^ produces equivalent values of −10.4, −15.7, and −20.8 pA, comparable to the current levels in the basal OHC of Fig. 5c.

**Table 1:** Single-channel current levels in outer hair cells at different cochlear locations

Cochlear location	Level 1	Level 2	Level 3	Level 4	Level 5	*N* traces
Apex	−3.8 ± 0.5 (9)	−7.0 ± 0.8 (16)	−11.1 ± 1.1 (13)	−14.2 ± 1.6 (2)		29
Middle	−3.7 ± 0.8 (4)	−7.2 ± 0.9 (9)	−11.2 ± 1 (17)	−14.3 ± 1 (9)		32
Base		−6.2 ± 0.7 (5)	−11.3 ± 0.9 (13)	−14.7 ± 1.1 (17)	−20.7 ± 1.3 (19)	30

Each entry gives mean ± SD of the current level in pA extracted from amplitude histograms of individual traces (Fig. 5), with the number of occurrences in parentheses. Recordings in *Tmc2*^*−/−*^ mice at −84 mV holding potential, with stereociliary bundles exposed to saline containing 0.04 mM Ca^2+^

The similarity of the current levels across cochlear regions suggests that only a discrete number of channel conductance states, possibly five or six, are required to account for the tonotopic variation. To derive an estimate of the “effective” unitary channel conductance, the weighted average channel conductance, *g*_wav_, for each cell was calculated (see Methods). *g*_wav_ (mean ± SD) was 88 ± 11 pS (*N* = 3; apex), 133 ± 17 pS (*N* = 3; middle) and 169 ± 26 pS (*N* = 4; base), increasing from apex to base (Supplementary Fig. 2), and therefore consistent with the previously determined tonotopic gradient (Fig. 2e). A similar two-fold gradient was seen in the median values of the distributions, with inferred mean conductances of 87 ± 5 pS (*N* = 3, apex), 142 ± 29 pS (*N* = 3, mid), and 177 ± 8 pS (*N* = 4, base). An explanation for these results is that each transduction complex contains multiple MET channels, and the number of channels increases along the cochlea. Measurements were also made on IHCs (Fig. 4b), and in two apical cells, *g*_wav_ was 96 pS, similar to that for apical OHCs.

One interpretation of these single-channel measurements is that there are more MET channels in basal OHCs than in apical OHCs. If this is the case, the pore itself may be the same at both locations. The alternate explanation would be that the pore at the base is different, enabling a larger current to flow through it. We examined this hypothesis by comparing the channel permeability at the two extreme locations in *Tmc2*^*−*^^*/−*^ mice. The reversal potential of the MET current when using *N*-methylglucamine (NMG) as the sole monovalent ion in the external solution was −49.7 ± 4.0 mV (*N* = 3) at the base and −51.3 ± 1.2 (*N* = 3) at the apex. These give permeability ratios of NMG relative to Cs^+^ of 0.144 ± 0.02 (*N* = 3; base) and 0.135 ± 0.01 (*N* = 3; apex), values that are not significantly different (*P* = 0.55). The Ca^2+^ permeability of OHC MET channels in *Tmc2*^*−/−*^ mice was also similar at the two locations, with Ca^2+^ permeability relative to Cs^+^ permeability of 3.9 ± 0.3 (*N* = 5; base) and 4.2 ± 0.2 (*N* = 5; apex).

### TMC1 and TMC2 are expressed in a tonotopic gradient

**Fig. 6:**
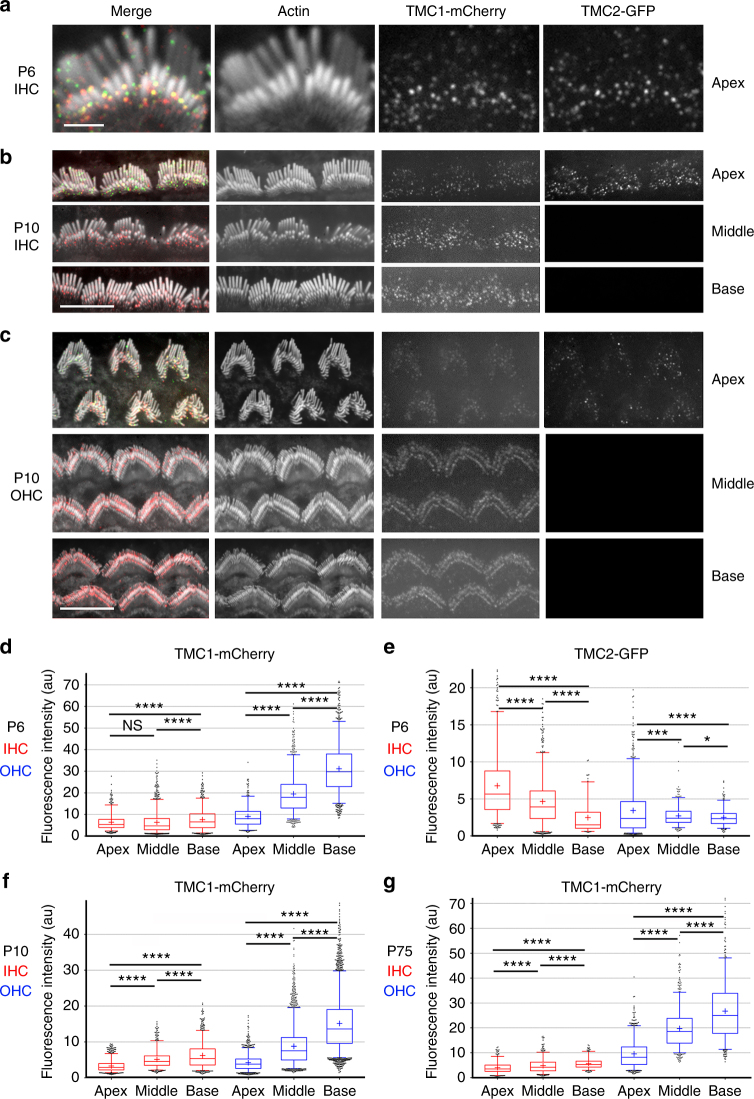
TMC levels in stereocilia show tonotopic gradients. **a** Leftmost panel, IHC stereocilia (stained for actin with Alexa-405 phalloidin, shown in white) from the apical cochlear turn from a P6 mouse expressing TMC1-mCherry (red) and TMC2-GFP (green). Subsequent panels show individual channels, respectively: actin (white), TMC1-mCherry (red), and TMC2-GFP (green). Scale bar, 2 µm. **b** Confocal images of IHC bundles from P10 transgenic mouse showing that TMC1-mCherry is expressed throughout the cochlea (apex, middle, and base) but TMC2-GFP expression is restricted to an apical cochlear location, scale bar = 10 µm. **c** Confocal images of hair bundles from P10 transgenic mouse showing expression of TMC1-mCherry from an apical, middle, and basal cochlear location, TMC1 expression increasing from apex to base, scale bar = 10 µm. TMC2-GFP is only detected in apical OHCs. Box whisker plots of TMC1-mCherry puncta fluorescence intensity in P6 female (**d**), P10 male (**f**) and P75 female (**g**) mice, increasing almost three-fold from apex to base in OHCs at all ages, but only slightly in IHCs. **e** TMC2-GFP expression decreases from apex to base in P6 IHCs, and shows a relatively smaller decrease in OHCs. Box plot: Boxes represent 1st and 3rd quartile; central line depicts data median; “+” represents the mean; whiskers depict 5–95 percentile; and data points beyond the whiskers are outliers beyond the 5 or 95 percentile of all data. ****, *p*-value < 0.0001; ***, *p*-value < 0.001; *, *p*-value < 0.05. (Student’s *t-*test)

To relate the single MET channel measurements to the stereociliary distribution of TMC1 and TMC2, we used transgenic mice on a *Tmc1*^−/−^:*Tmc2*^−/−^ background, expressing TMC1 and TMC2 fused at their C-termini to fluorescent tags: *Tmc1-mCherry* or *Tmc2-GFP*^15^. Female mice showed mosaic expression of *Tmc1-mCherry* and *Tmc2-GFP*, so that hair cells within the same cochlea expressed TMC1-mCherry or TMC2-GFP, or neither or both^15^. We previously showed that TMC1-mCherry and TMC2-GFP express as discrete puncta at the stereocilia^15^. Puncta were more widely distributed along the length of the stereocilia early postnatally, and by P6 both TMC1 and TMC2 are largely present at the sites of the MET channels at tips of the second row and lower stereocilia in apical hair cells (Fig. 6a). Importantly, because the transgenic mice expressing TMC1-mCherry and TMC2-GFP had a *Tmc1*^*−/−*^*:Tmc2*^*−/−*^ background, all the TMC1 and TMC2 expressed in these mice were fluorescently tagged^15^. We cannot exclude the possibility that TMC1-mCherry and TMC2-GFP are overexpressed in the hair cell, or their function affected by the fluorescent tags. However, as previously reported^15,16^, the mice showed normal hearing thresholds, vestibular function, and whole-cell MET currents, indicating that the fluorophore-tagged proteins are functional and properly targeted to the MET complex site. The fluorescence intensity measured in the red or green channel thus correlates directly with the number of TMC1-mCherry or TMC2-GFP molecules, respectively. To examine whether the number of TMC molecules at each MET site varies along the cochlea, we measured the relative fluorescence intensity (RFI) per punctum in stereociliary bundles of hair cells from the apex, middle and base, in hair cells expressing both isoforms. At P6, ^15^ (Fig. 6a), the RFI (mean ± SD in arbitrary units, AU; *N* = number of fluorescence puncta) of TMC1-mCherry puncta in stereocilia showed a linear, ~3-fold increase from apex to base in OHCs (apex 9.1 ± 5, *N* = 461; middle 19.5 ± 9, *N* = 1032; base 31.2 ± 12, *N* = 1174) with only a small increase in IHCs (apex 6.4 ± 3.8, *N* = 557; middle 6.4 ± 5, *N* = 1315; base 7.6 ± 5, *N* = 766) (Fig. 6d; Supplementary Fig. 3). The RFI of TMC2-GFP puncta in stereocilia at P6 (Fig. 6e) exhibited a significant change in the opposite direction to the TMC1-mCherry gradient in IHCs (apex 6.8 ± 5, *N* = 567; middle 4.7 ± 3, *N* = 817; base 2.5 ± 2, *N* = 147) and in OHCs (apex 3.4 ± 3, *N* = 750; middle 2.7 ± 1.4, *N* = 398; base 2.5 ± 1, *N* = 275). This is probably due to the decline in TMC2 expression^12^, which begins at this age and proceeds from base to apex. Stereociliary expression of TMC2-GFP was no longer detected in IHCs or OHCs of the cochlear middle turn after P8^15^, but was still evident at the apex at least up to P10 (Fig. 6b, c). There was considerable variability in the RFI of the puncta, even at P10 when they localized primarily to the MET channel site (Fig. 6c). However, the TMC1-mCherry tonotopic gradients in mean RFI, consistently smaller across IHCs and larger across OHCs (Fig. 6f, g), were maintained at P10 (IHCs: apex 3.3 ± 1.6, *N* = 972, middle 5.2 ± 2.5, *N* = 662; base 6.3 ± 3.5, *N* = 951; OHCs: 4.2 ± 2.3, *N* = 1332; middle 8.8 ± 5.4, *N* = 2874; base 15.2 ± 7.9, *N* = 3248) and also in the mature P75 cochlea (IHCs: apex 4.0 ± 2.4, *N* = 332; middle 4.8 ± 2.9, *N* = 496; base 5.7 ± 2.2, *N* = 286; OHCs apex 9.6 ± 5.5, *N* = 824; middle 19.8 ± 7.9, *N* = 797; base 26.8 ± 11.9, *N* = 566). These data parallel the two to three-fold increase in TMC1-dependent single-channel conductance in OHCs along the tonotopic axis at P5-6, with no major increase in the TMC2-dependent channel conductance (Fig. 2e).

### Estimation of the number of TMC molecules per MET complex

**Fig. 7:**
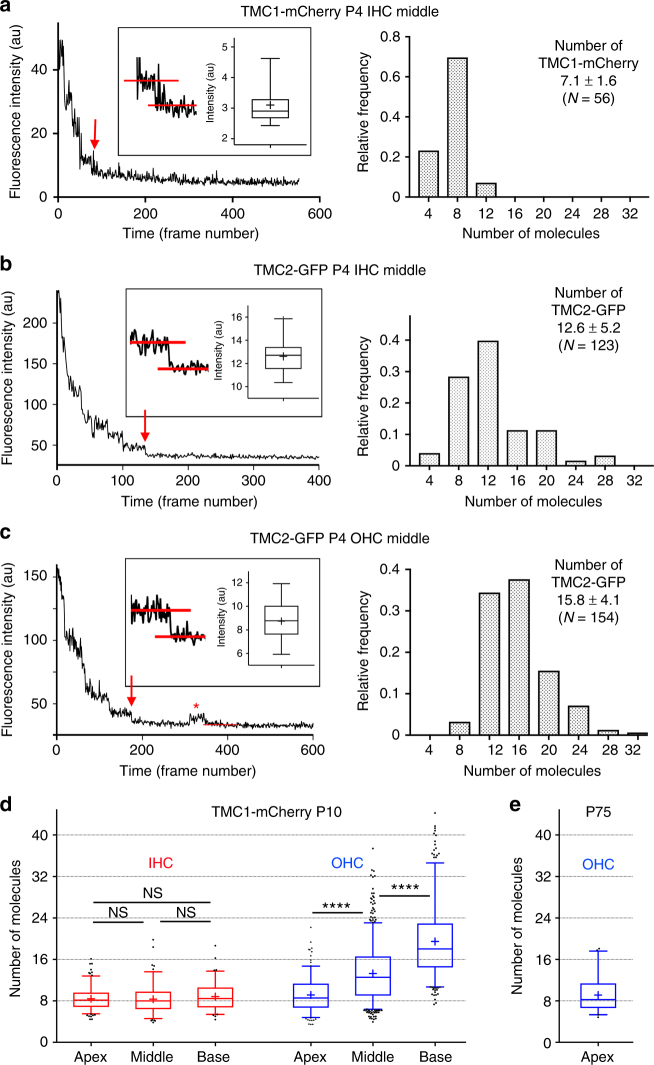
Bleaching and blinking steps of single TMC1-mCherry and TMC2-GFP puncta reveal the comprising number of molecules. **a** Left panel: time course of maximum fluorescence intensity of TMC1-mCherry puncta in IHC stereocilia at P4. Red arrow points to last bleaching step. Inset: box plot showing fluorescence intensity (mean ± SD) of final bleach step. Right panel: frequency distribution of number of TMC1-mCherry molecules within each fluorescent punctum. Mean of distribution ± SD. **b**, **c** Left panel: time course of integrated fluorescence intensity of TMC2-GFP puncta in IHC stereocilia at P4 (**b**), and TMC2-GFP puncta in OHC stereocilia at P4 (**c**). Red arrows point to final bleaching step. Inset: Box whisker plot showing fluorescence intensity (mean ± SD) of final bleach step. Asterisk highlights occasional blink after the GFP had reached the dark state. Right panels: frequency distribution of number of TMC2-GFP molecules within each fluorescent punctum. **d** Number of TMC1-mCherry molecules per fluorescence punctum increases from apex to base in OHC stereocilia at P10, but not in IHC stereocilia; number of measurements: IHC apex, *N* = 177; middle, *N* = 118; base, *N* = 99; OHC apex, *N* = 211; middle, *N* = 656; base, *N* = 355. **e** Number of TMC1-mCherry molecules per fluorescence punctum in apical OHC stereocilia at P75, *N* = 53. Box plot: whiskers, 5–95 percentile; +, mean. ****, *p*-value < 0.0001. (Student’s *t-*test; number of measurements indicated on plots; NS not significant)

We next estimated the number of fluorescent molecules in each punctum based on the bleaching (and blinking) behavior of the fluorophore. The number of fluorescently-tagged molecules per punctum was obtained by dividing the RFI of each punctum at time zero by the value for a single fluorophore obtained by analyses of fluorescence decay curves (Fig. 7a–c, left panels) (see Methods). Histograms of the frequency distribution vs. estimated copy number are shown in Fig. 7a–c, right panels. The mean number of TMC1-mCherry per punctum in middle turn IHCs at P4 was calculated to be 7.1 ± 1.6 (*N* = 56); TMC2-GFP per punctum in IHCs was 12.6 ± 5.2 (*N* = 123); and TMC2-GFP molecules per punctum in middle turn OHCs was 15.8 ± 4.1 (*N* = 154). The number of TMC1-mCherry molecules at MET sites was then estimated along the tonotopic gradient of IHCs and OHCs in fully developed stereociliary bundles, when TMC2 is no longer expressed. At P10, when apical IHCs still express some TMC2, the number of TMC1-mCherry molecules at the MET site in IHCs was 8.4 ± 2.1 (*N* = 177; apex), 8.3 ± 2.8 (*N* = 118, middle turn), and 8.8 ± 2.7 (*N* = 99, base). The TMC1-mCherry numbers in OHCs were 9.1 ± 3.2 (*N* = 211, apex), 13.3 ± 5.4 (*N* = 656, middle) and 19.5 ± 6.9 (*N* = 355, base) (Fig. 7d). The number of TMC1-mCherry molecules in apical OHCs of adult mice at P75 (Fig. 7e) was 9.1 ± 3.2 (*N* = 53), consistent with the value at P10. It should be noted that these numbers are likely to be an underestimate as it has been shown that ~20% of GFP molecules are non-fluorescent due to misfolding^32,33^.

### TMC1 and TMC2 required for stereocilia staircase development

**Fig. 8:**
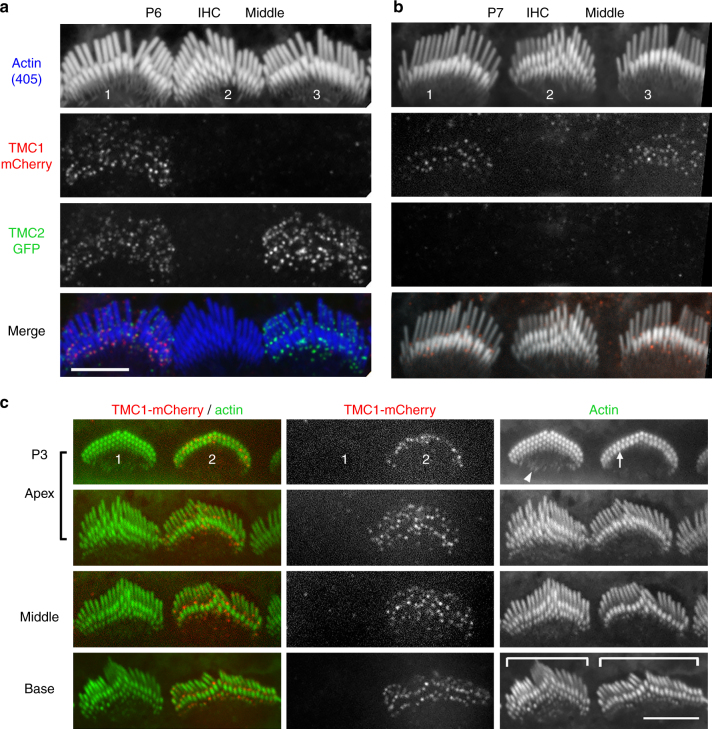
TMC1 and TMC2 are required for normal stereocilia bundle development and morphology. **a**, **b** Confocal images of three IHC stereocilia bundles (1–3) from cochlear middle turn labeled with phalloidin (top panel), from transgenic mice aged P6 (**a**) and P7 (**b**), with a mosaic expression of TMC1-mCherry (second panel), and/or TMC2-GFP (third panel). Bottom panel—merge image of all three channels. Stereocilia bundles of IHCs that do not express TMC1-mCherry or TMC2-GFP (e.g., bundle 2 in **a** and **b**) are clearly developmentally immature relative to IHC bundles expressing either TMC1-mCherry or TMC2-GFP. **c** Confocal images of two adjacent IHC bundles from the cochlear apex (top two panels), middle (third panel) and base (fourth panel), from transgenic mice aged P3, with a mosaic expression of TMC1-mCherry, and no TMC2-GFP. One IHC lacks both TMCs, and the second expresses only TMC1-mCherry. Note that in the absence of TMC1 and TMC2, stereociliary bundles have extra rows and morphology remains similar at the apex, middle and base. Conversely, expression of TMC1-mCherry is necessary and sufficient for the normal reduction in number of stereocilia rows, and the subtle widening of the angle of the bundle “V”-shape from apex to base. Scale bar = 5 µm

MET function, directly or indirectly, regulates stereociliary properties^19,22,21^. To investigate contributions of TMC1 and TMC2 to hair bundle structure, we used the *Tmc1*^*−/−*^*:Tmc2*^*−/−*^ mice with mosaic expression of *Tmc1-mCherry* and *Tmc2-GFP*, so that hair cells within the same cochlea expressed TMC1-mCherry or TMC2-GFP, or neither or both, providing side-by-side controls along the tonotopic gradients of the cochlea. The presence of either TMC1-mCherry (Fig. 8a, bundle 1) or TMC2-GFP (Fig. 8b, bundles 1 and 3) correlates with a normal IHC hair bundle morphology, with two rows of tall stereocilia and third and fourth rows of shorter and thinner ones at P6 (Fig. 8a, bundle 1). Stereociliary bundles from IHCs lacking both TMC1 and TMC2 showed an immature phenotype, consistent with delayed or arrested development, including multiple rows of stereocilia forming a staircase with regular step size, organized in a pyramidal shape (Fig. 8a, b, bundle 2). Maturation of hair cells at the base of the cochlea precedes the apex by about 2 days^6^. To further examine the role of TMC1 in stereociliary bundle development, we imaged IHC stereociliary bundles at P3, along the tonotopic axis (Fig. 8c). In IHCs lacking both TMC1 and TMC2, the bundle morphology was comparably immature at apex, middle, and basal turns (Fig. 8c, bundle 1). Strikingly, the expression of TMC1-mCherry alone was sufficient to produce normal hair bundle development along the cochlea (Fig. 8c, bundle 2). These data suggest that expression of either TMC1 or TMC2 is required for stereociliary bundle development.

The absence of both TMC isoforms from OHCs at P6 resulted in stereociliary bundles with multiple rows (Supplementary Fig. 4), and a change in the morphology from an open “V-shape” to a closed “U-shape” (Supplementary Fig. 4a–c, bundle 2). Such rounded bundles have been previously seen in *Tmc1*^*−/−*^*: Tmc2*^*−/−*^ mice^12,29^ as well as in other mutants linked with loss of mechanotransduction^34^.

### TMC2 expression in IHC stereocilia depends on TMC1

**Fig. 9:**
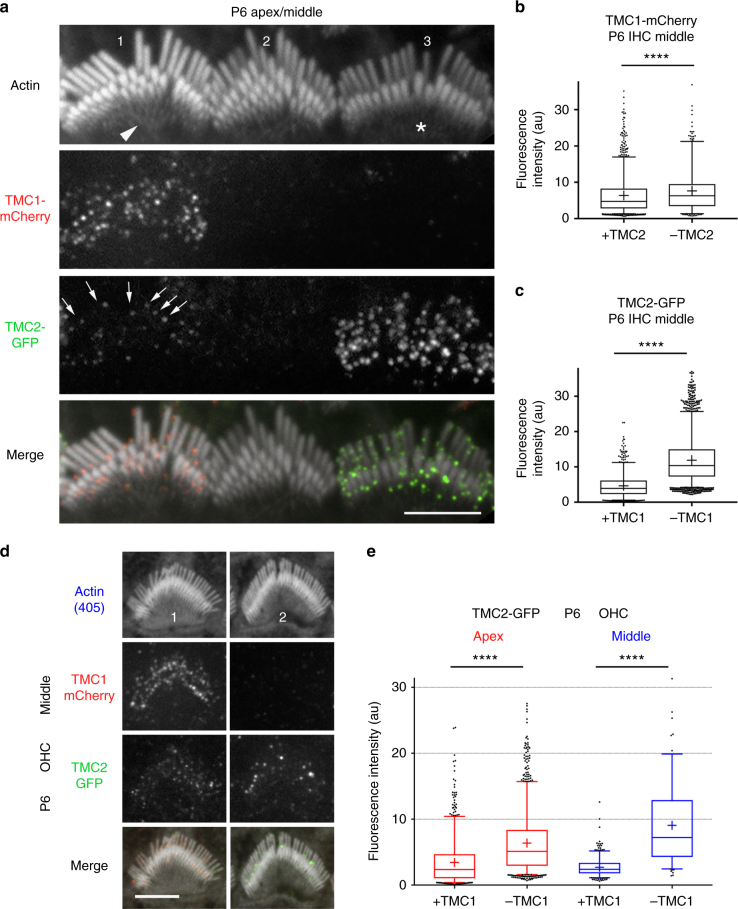
TMC2-GFP levels in IHC and OHC stereocilia are influenced by TMC1-mCherry levels. **a** Confocal image of three IHC stereociliary bundles (bundles 1–3) from a P6 transgenic mouse with a mosaic expression of TMC1-mCherry (second panel), and/or TMC2-GFP (third panel). Some cells do not express any transgene (e.g., bundle 2). Bottom panel, merge image of all three channels. TMC2-GFP levels are comparatively lower (arrows in bundle 1) in the presence of TMC1-mCherry than in the absence of TMC1-mCherry (bundle 3). Note that relative to normal bundle morphology when both TMC1-mCherry and TMC2-GFP are expressed (bundle 1), bundle morphology is immature, with multiple stereocilia rows, when both TMC1 and TMC2 are missing (bundle 2), and more developed when TMC2-GFP is more highly expressed in the absence of TMC1-mCherry. **b** Relative fluorescence intensity of TMC1-mCherry puncta in IHC stereocilia at P6 in IHCs that express TMC2-GFP is slightly lower (6.4 ± 0.1 arbitrary units; *N* = 1315), compared with IHC that do not express TMC2-GFP (7.6 ± 0.3; *N* = 814). **c** Relative fluorescence intensity of TMC2-GFP puncta in IHC stereocilia at P6 increases significantly in IHCs that do not express TMC1-mCherry (11.9 ± 1.4; *N* = 2134), compared with IHCs that do express TMC1-mCherry (4.7 ± 0.1; *N* = 814). **d** Confocal images of two OHC stereocilia bundles (#1–2) from cochlear middle turn from transgenic mice (P6) with a mosaic expression of TMC1-mCherry (second panel), and/or TMC2-GFP (third panel). Bottom panel—merged image of all three channels. Note that TMC2-GFP puncta are brighter in absence of TMC1-mCherry. **e** Plot of relative fluorescence intensity of TMC2-GFP puncta in P6 OHCs at cochlear apex in presence (3.4 ± 1.2; *N* = 750) and absence (6.4 ± 1.3; *N* = 1252) of TMC1-mCherry; and at middle turns in presence (2.7 ± 0.1; *N* = 398) and absence (9.1 ± 0.5; *N* = 1151) of TMC1-mCherry. Scale bar = 5 µm. Box plot: whiskers, 5–95 percentile; +, mean. ****, *p*-value < 0.0001 (Student’s *t-*test; all values expressed as mean ± SD)

By using transgenic mice with mosaic expression of TMC1-mCherry and TMC2-GFP in P6 IHCs, we could determine the expression of one TMC isoform in the absence of the other. The expression of TMC2-GFP within the stereociliary bundle was higher when hair cells did not express TMC1-mCherry (Fig. 9a, bundle 3, Fig. 9c) than when both isoforms were present (Fig. 9a, bundle 1, Fig. 9c). The lack of TMC2-GFP also led to a slight increase in TMC1-mCherry levels (Fig. 9b). These data suggest removal of TMC2 from stereocilia by P7 may be facilitated by the increased levels of TMC1 at this age. A significant increase in TMC2-GFP expression was also observed in stereocilia of OHCs lacking TMC1-mCherry (Fig. 9d, bundle 2, 9e) relative to OHCs expressing both isoforms (Fig. 9d, bundle 1, 9e). By P6 the overexpressed TMC2-GFP appeared as fewer and larger fluorescent puncta (Fig. 9d, bundle 2). We found that TMC2-GFP expression in basal OHCs stereocilia, when TMC1-mCherry was also expressed, was significantly lower compared to the middle (Supplementary Fig. 4b and c), depicting the beginning stages of TMC2 removal. In absence of TMC1-mCherry, TMC2-GFP was still significantly upregulated (Supplementary Fig. 4c), the TMC2-GFP fluorescence puncta being fewer but brighter. We cannot distinguish whether these brighter puncta were preexisting and were not removed as efficiently as the smaller TMC2-GFP clusters, or whether TMC2 molecules assembled into larger aggregates during removal from the stereocilia.

A notable effect of overexpression of TMC2 in the absence of TMC1 was an apparent acceleration in bundle development. This phenotype is exemplified in Fig. 9a, where bundle 3 (lacking TMC1-mCherry and expressing higher levels of TMC2-GFP) has fewer rows of stereocilia and a more mature phenotype than bundle 1 (expressing both isoforms). It is also important to note that in such hair bundles, TMC2-GFP is highly expressed in the shortest rows of stereocilia that are pruned during maturation. These results are consistent with the MET current measurements where absence of TMC1 prolonged expression of TMC2 (Fig. 1e). They also support differential roles for the two isoforms in bundle development.

## Discussion

TMC1 and TMC2 isoforms have disparate periods of expression, TMC2 occurring transiently after birth, and TMC1 emerging later^12,15^. We highlight the functional differences between the two isoforms and examine their relative contributions to the spatiotemporal development of the MET channel properties and stereocilia staircase organization. Channels containing TMC1, compared to TMC2, show a larger average single-channel conductance at all cochlear locations and a two-fold apical-basal gradient in channel conductance in OHCs, for which TMC2 is not required (Fig. 2). Since our hypothesis (see below) is that larger conductance channels are generated by cooperative opening of smaller channels, this suggests that the lowest level (50 pS) is larger for TMC1 than TMC2, and moreover TMC1 may be more cooperative. If the difference in Ca^2+^ permeability between the two isoforms^5^ is taken into account, multiple aspects of ion conduction through the MET channel depend on which TMC isoform is present, consistent with the notion that TMC1 and TMC2 can influence the MET channel pore^5,16^.

We also show that the presence of TMC1 alone enables an apical-basal gradient in unitary conductance, which, together with an increase in the number of stereocilia per OHC bundle^28,35^, augments the hair-cell MET current several fold. The larger basal OHC current will enhance the sensitivity in high-frequency OHCs^36^. Using transgenic mice expressing TMC1-mCherry and/or TMC2-GFP we found, consistent with the physiology data, that the expression level of TMC1-mCherry at the site of MET in OHCs follows a tonotopic gradient along the length of the cochlea, increasing from apex to base, with a smaller increase in IHCs. These data suggest that increasing levels of TMC1 at the MET channel site are the major contributor to the apical-basal gradient in channel conductance. We therefore propose that the number of TMC1-dependent channels per MET complex, rather than TMC1/TMC2 stoichiometry as previously suggested^16^, underlies the tonotopic conductance gradient.

If TMC1 is the only TMC isoform in mature MET channels of all OHCs, then what accounts for the difference in channel conductance in apical and basal OHCs? New information about the origin of tonotopy has been derived by analyzing single-channel conductance by recording under conditions that enhanced the signal to noise ratio of whole-cell recordings. Strikingly, we observed increased levels of current at approximately equal increments of ~3.7 pA, corresponding to levels of 7.4, 11.1, 14.8 pA, and one at a slightly larger interval at 20 pA. The approximate agreement of these conductance levels across cochlear regions suggests that only a small number of levels, possibly five or six, are required to cover the tonotopic gradient. This stepwise tonotopic gradient raises the possibility that the tonotopic variation involves increasing the number of small-conductance channels per MET site. It must be stressed that these experiments do not define the location of the different levels, and the analysis cannot distinguish between multiple conductance states of an MET channel complex in one stereocilium, and the presence of channels with different current amplitudes in different stereocilia. Previous electrophysiology and modeling data have suggested more than one channel per MET site^28,37^. The observed branching of the tip-link protein PCDH15 into two or three strands at the site of insertion at the lower tip-link insertion site^38,39^ agrees with the possibility that the tip-link is attached to more than one channel at the MET site. Consistent with the hypothesis of multiple channel units per MET site, the relative expression of TMC1-mCherry in OHCs increases about three-fold from apex to base, whereas in IHCs, there is only small increase comparable to the small gradient in IHC channel conductance. Estimates of the number of TMC1-mCherry per MET site using the bleaching properties of the fluorophore show an average of ~8–20 TMC molecules per MET site in OHCs depending on their cochlear localization, while only ~8 TMC molecules are observed in IHCs across the tonotopic gradient, paralleling the gradients of conductance levels.

The results taken together suggest each MET channel complex contains multiple TMC1 molecules, and that there are varying numbers of channels per MET site. However, they provide no insight into how multiple molecules operate in a coordinated manner. One idea is that the transduction complex comprises multiple channels, but the force input opens the channels in a coordinated or cooperative manner. Thus, opening of one channel in the transduction complex by applied force might rapidly facilitate opening of others, so the current transition appears to be that of a single larger channel. Such cooperativity has been hypothesized for Slack K^+^ channels^40^ and may also arise due to elastic forces causing an interaction between channels through the lipid bilayer^41,42^. It is not yet known whether TMC proteins make up the pore component of the channel, nor is it clear how many TMC molecules are associated with each unitary channel. While our data highlight a direct correlation between the number of TMC1 and TMC2 proteins and MET current, we still cannot rule out that these proteins may play an auxiliary role. Recent reports also suggest that MET function is dependent or regulated by other stereociliary proteins including TMIE^14^, LHFPL5^10,13^, and Cib2^34^.

We also show that development of the stereociliary bundle depends on the presence of either or both TMC isoforms, highlighting a link between TMC expression, MET activity, and stereociliary length regulation during bundle development. Specifically, stereocilia bundles from hair cells lacking both TMC1 and TMC2 showed a phenotype consistent with delayed development, including multiple rows of stereocilia forming a staircase with regular steps and a symmetric pyramidal organization of the tallest stereocilia in the axis orthogonal to the staircase. Expression of either TMC1 or TMC2 at this stage was sufficient for the progression of the development of bundle morphology. Strikingly, however, the overexpression of TMC2 (in the absence of TMC1), appeared to accelerate bundle development with faster resorption of the supernumerary microvilli. Thus, TMC1 and TMC2 are likely to make differential contributions toward MET channel properties, as well as the complex mechanisms that regulate bundle development and the slope of the stereocilia staircase. It is noteworthy that while the mean (and median) number of TMC molecules per punctum at stereocilia MET sites are remarkably consistent at each tonotopic location (Figs. 6 and 7), there is a large variability within each stereociliary bundle. This may be the result of stochastic variations in the trafficking and retention of TMCs at the MET complex at each stereocilium. We do not know how this stochastic variation locally affects stereocilia length regulation. It has been shown that slight and transient differences exist in the heights of individual stereocilia in the second and third rows^19,22^. We observed that in the basal cochlear turn at P6, when TMC2 levels start to decline, the removal of TMC2-GFP occurred non-uniformly, with fewer, brighter GFP fluorescence puncta, that are probably aggregates of TMC2-GFP, retained at the tips of the stereocilia. The presence or absence of TMC2-GFP, besides affecting individual stereocilia, must have an influence on bundle morphology. It is therefore likely that the TMCs influence a yet-to-be-determined pathway regulating overall bundle development and degeneration. Both MET current recordings and expression of TMC isoforms indicate that in the absence of TMC1, TMC2 expression is increased and prolonged suggesting that: (i) the decreasing levels of TMC2 from stereocilia which normally begins at approximately P6-P7 may be promoted by the increasing levels of TMC1; and (ii) in the absence of TMC1, TMC2 is either upregulated or retained for a longer time at the MET site. However, there is no evidence that TMC2 rescues hearing in adult mice lacking TMC1^12^.

## Methods

### Mouse mutants

The care and use of animals for all experiments described conformed to NIH guidelines, and were approved by the Institutional Animal Care and Use Committees at the University of Wisconsin-Madison IACUC, and at the National Institute on Deafness and Other Communication Disorders (protocol #1215). For electrophysiology experiments, the *Tmc1* mutant used was B6.129-*Tmc1*^*tm1.1Ajg*^/J^12^, obtained from Jackson Labs (stock number 019146); the *Tmc2* mutant (B6.129S5-*Tmc2*^*tm1Lex*^/Mmucd) was obtained from the Mutant Mouse Regional Resource Center, University of California, Davis, CA. As discussed previously^17^, the two mutants were effective nulls. A mixture of male and female mice was used and no gender-specific effects were noted. Both B6 heterozygotes and outbred ICR mice were used as controls. For fluorescence localization experiments, the two TMC isoforms were visualized in hair cells of transgenic mice, obtained from Dr. Andrew Griffith, NIDCD^15^, expressing TMC1 or TMC2 fused at their C-terminus to fluorescent tags: *Tmc1-mCherry* or *Tmc2-AcGFP*, on a *Tmc1*^*−/−*^*:Tmc2*^*−/−*^ background, such that all TMC1 and TMC2 expressed were fluorescently tagged. Mice were genotyped using primers recognizing unique sequence within mCherry:

Tmc1mCherryL 5′-TTCACTTGCCCTTCTTCATCTC, Tmc1mCherryR 5′-CGCCCTCGATCTCGAACT; and AcGFP: Tmc2AcGFPL 5′-ACCATGTTGGGTCTCAACCAC, Tmc2AcGFPR 5′-TGAACTTGTGGCCATTCACAT. As described previously^15^, female mice from *Tmc1-mCherry* line 3 showed a mosaic expression of TMC1-mCherry in IHCs and OHCs, most likely because the bacterial artificial chromosomes (BACs) encoding the Tmc1-mCherry was integrated into the X chromosome, and therefore underwent X-linked inactivation in ~50% of cells. To obtain mice expressing both fluorescently-tagged isoforms, we subsequently crossed mice from *Tmc1-mCherry* line 3 with multiple *Tmc2-AcGFP* lines. Serendipitously, we generated a unique line in which IHCs and OHCs expressed: (1) both TMC1-mCherry and TMC2-AcGFP (which we refer here as TMC2-GFP), (2) only TMC1-mCherry, (3) only TMC2-GFP, or (4) neither TMC isoform. While we cannot be certain of the genetic underpinnings that caused this mosaic expression of all combinations of TMC expression, one possibility is that the *Tmc2-GFP* was also integrated in the X-chromosome, but the two genes were not closely linked. X-chromosome recombination during cell division, early during the development of the cochlea, then resulted in daughter cells with X-chromosomes containing various combinations of *Tmc1-mCherry* and/or *Tmc2-GFP*, as the cochlea extended. This expression pattern allowed us the remarkable opportunity to directly assess the effects of expression of either, both or neither TMC isoforms, in hair cells that were side-by-side within the same cochlea.

### Electrophysiology and stimulation

MET currents were recorded from OHCs and IHCs in isolated organs of Corti of mice between embryonic day (E) 18 and postnatal day 16 (E18 to P16, where E19 = P0 is the birth day) using methods described previously^5,17^. In order to document developmental changes, serial measurements were made on pups from a given litter at different stages of development, 24 h apart. Results were then collated from three or more separate litters. The recording and stimulation methods were identical to those previously described^17,29^. Excised cochlear turns were immobilized in a recording chamber on a fixed-stage microscope (Leica DMFS), and viewed through a 63× long working distance water-immersion objective. The recording chamber was perfused with saline of composition (in mM): 152 NaCl, 6 KCl, 1.5 CaCl_2_, 2 Na-pyruvate, 8 d-glucose and 10 Na-HEPES, pH 7.4, at room temperature, 21–23 °C. Electrical recordings were made with borosilicate patch electrodes, filled with a solution (in mM): 128 CsCl, 3.5 MgCl_2_, 5 Na_2_ATP, 10 Tris phosphocreatine, 1 EGTA, 10 Cs-HEPES, pH 7.2, and connected to an Axopatch 200B amplifier. Voltage-clamp protocols were usually referred to a holding potential of −84 mV. MET channel permeability to *N*-methylglucamine (NMG) was determined from measurements of reversal potentials with 160 mM NMG replacing Na^+^ and K^+^ in the external medium, corrected for a junction potential of 10.5 mV. Hair bundles were stimulated using a fluid jet^5^ from a pipette, with a tip diameter of 10–15 μm, driven by a 25-mm diameter piezoelectric disc. The distance of the pipette tip from the bundle was adjusted to elicit a maximal MET current. During fluid-jet stimulation, bundle motion was monitored by projecting an image of the bundle tip onto a pair of photodiodes (LD 2–5; Centronics) at a total magnification of 340 and measuring the differential photocurrent, which was calibrated by displacing the photodiodes a known distance in the image plane. The validity of the method has been substantiated by simultaneous high speed imaging^10^. No difference was seen in motion elicited in wild-type, *Tmc1*^*−/−*^ and *Tmc2*^−/−^ mice.

Single MET channels were characterized from whole-cell recordings after brief exposure to submicromolar Ca^2+^ buffered with 4 mM BAPTA^17,28^. Currents were filtered at 3 kHz with an 8-pole elliptic filter and digitized at 100 kHz. IGOR Pro v6 (Wavemetrics, Lake Oswego, OR) was used to generate amplitude histograms of the current levels from ~100 to 200 sweeps of digitized data and fit with a pair of Gaussians. The possibility of multiple conductance states was investigated by patch recordings with bundles exposed to low (0.04 mM) extracellular Ca^2+^, which increases the MET channel amplitude^9^ and slows the kinetics, both factors augmenting the signal-to-noise ratio. In the best cases, the noise was less than 2 pA r.m.s. for 3 kHz bandwidth, being limited by electrode series resistance (<5 Mohms) and hair-cell capacitance, and was independent of extracellular Ca^2+^. In these experiments, current records were analyzed by manual inspection of each trace for discrete current levels lasting 1 ms or more, and quantifying each level after zeroing a short sequence prior to the opening transition. Prolonged openings were counted as multiple events so the amplitude of the histogram reflected the duration that channel component was present. Amplitude histograms were also constructed from individual traces and the channel current levels averaged across the array of sweeps for a given cell. The experimental measurements and channel analysis were done blind by different investigators prior to genotyping. Current events were verified as MET channels because they were abolished by perfusion with 0.1 mM dihydrostreptomycin. Amplitude histograms were constructed for 200–400 channel events, and were fit with a sum of Gaussians. For a given cell, the weighted average channel conductance, *g*_wav_, was calculated by: *g*_wav_ = Σ (*i*_*i*_.*n*_*i*_.*σ*_*i*_)/Σ (*n*_*i*_.*σ*_*i*_)/V_H_, where *i*_i_ is the peak current of the *i*th Gaussian in the fit and *n*_*i*_ is the amplitude and *σ*_*i*_ is the standard deviation of the Gaussian fit, and *V*_H_ is the holding potential (−84 mV); *n*_*i*_ is a measure of the time that that channel component was present. Unless otherwise stated, all results are quoted as mean ± 1 standard deviation (SD), and statistical significance is assessed using a two-tailed Student’s *t*-test. For each type of experiment, the sample size was determined based on previous results. G-Power analysis was performed to estimate the number of animals to achieve a given signal-to-noise ratio of 1–1.5, with 80% power at a 5% significance level. The development of the MET current, *I*, against postnatal days, *d*, was fit with a sigmoidal relation: *I* = *I*_max_/ (1 + exp ((*d*_0.5_ − *d*)/*d*_s_)), where *I*_max_ is the maximum current, *d*_0.5_ is the half-maximal time, and *d*_s_ is the slope factor.

### Fluorescence microscopy

TMC1-mCherry and TMC2-GFP were detected and localized using spinning disk confocal fluorescence microscopy as previously described^15^. Briefly, cochlear tissue from mice expressing TMC1-mCherry and TMC2-AcGFP was rapidly isolated and fixed in 4% formaldehyde in Leibovitz L-15 medium for 20 min and then washed with phosphate-buffered saline (PBS). Samples were then permeabilized with 0.5% Triton X-100 in PBS containing Alexa Fluor 405 phalloidin (to label stereociliary actin) for 30 min, before washing and mounting between a microscope slide and cover slip. Samples were viewed in a Nikon inverted fluorescence microscope, fitted with a spinning disk confocal scan head (Yokogawa, CSU-22), 100 × Apo 1.49 numerical aperture objective, and an EM-CCD (Andor 888 or 897) camera. Total magnification was 40 or 60 nm per pixel to accommodate the high dynamic range of the fluorescence signal^43^. Nikon NIS-Elements imaging software was utilized for image acquisition and analysis. For quantifying the fluorescence intensity of each TMC1-mCherry or TMC2-GFP puncta, the “spot detection” function of the NIS-Elements software was used to first select puncta within a 0.33 μm diameter ROI (the diffraction limit estimated for our optical setup). The “integrated fluorescence intensity” within the ROI was then used as the relative fluorescence intensity (RFI) of each punctum. Since each TMC1 protein is fused with one fluorophore, the integrated intensity of each diffraction-limited fluorescence punctum at stereocilia tips reflects, in principle, the number of tagged TMC1 molecules within that punctum. For internal consistency, measurements were made from the two organs of Corti per mouse fixed, counterstained, and mounted on the same slide and imaged under exactly the same acquisition parameters of the confocal microscope. Comparisons of RFI were made within each experimental condition and set of data for the two organs of Corti per mouse. Excel, Igor, and Prism software were used for statistical analyses and plotting of each data set as shown in the figures.

### Estimation of number of TMC molecules per fluorescence spot

Since each TMC protein is fused with one mCherry (or GFP) tag, the integrated intensity of each diffraction-limited fluorescent spot reflects the number of tagged TMC1 molecules within that spot. There are several known strategies for imaging and counting the number of fluorescent molecules in a single spot that are based on the blinking/bleaching behavior of fluorophores. Typically, these procedures use total internal reflection fluorescence (TIRF) microscopy for fast acquisition of a series of short exposures, at high signal to noise ratio, to allow the identification and counting discrete bleaching steps for individual fluorophores^32,44^. We could not use TIRF because we were imaging fluorescent molecules in a bulk tissue but instead used spinning disc confocal microscopy^45^. With the spinning disc, the fluorophores were exposed to light only for a fraction of the “exposure time” by the rotating pinholes, and only a fraction of the emitted light was collected by the spinning pinholes. This intermittence of the fluorescence extends significantly the lifetime as well as the blinking period of the fluorophores, but at the same time it reduces the emission rate and the fraction of the emitted light collected, thus requiring longer exposure times for optimal signal to noise ratio. We determined empirically that an optimal exposure time for each frame was 0.4–0.7 s in a running acquisition mode when combined with a ~15 mW laser power (for each of the 405, 488, and 561 nm lasers) at the fiber optic input to the spinning disk confocal scan head. We also determined empirically that for GFP fluorescence, using the integrated fluorescence intensity over the 0.33 µm diameter circular ROI resulted in the best signal to noise ratio, while for mCherry fluorescence, using the maximal value of fluorescence intensity produced better signal to noise ratio.

The plotting of the fluorescence intensity as a function of frame number showed a rapid decay due to the bleaching of the fluorophores (representative decay curves are shown in Fig. 7b). In order to estimate the number of TMC1-mCherry or TMC2-GFP molecules per MET site, we acquired a stream of images, and measured the integrated fluorescence intensity over the 0.33 nm diameter ROI for each fluorescence punctum in each sequential frame. The plot of fluorescence intensity as a function of frame number shows a rapid decay due to the bleaching of the fluorophores (representative decay curves are shown in Fig. 7a–c, left panels). Since the photobleaching of each fluorophore is a discrete process, the fluorescence intensity of a spot or punctum containing several fluorophores drops in a stepwise fashion, and the number of steps in principle reflects the number of fluorophores within the spot. However, when there are many fluorophores, the overlap of bleaching and blinking events within each acquired image frame made it difficult to discriminate single-molecule bleaching steps, except toward the end of the fluorescence decay curve when most of the fluorophores were bleached and only a few are left. We used the last step at the end of each fluorescence decay curve (Fig. 7a–c, arrows) as the value of fluorescence emission rate for a single fluorophore, or unitary emission rate. We only measured the last bleaching step in each decay curve when it was very distinct (e.g., a well-defined step after a distinct plateau above the background line, Fig. 7b), to minimize the possibility of including steps representing the overlap of bleaching of multiple fluorophores. We also occasionally observed “blinks” long after the fluorophores reached their dark state (Fig. 7c); the intensity of this blink, which was similar to the intensity of the last bleaching step, was also used as representative of the fluorescence intensity of a single fluorophore to compute the average unitary emission rate. The histogram of the fluorescent intensities of these single steps (last bleaching step and blinking events) exhibited a single Gaussian distribution, and we used the mean value as a measure of the integrated fluorescence intensity from one single mCherry molecule (e.g., Figure 7a). We then divided the fluorescence intensity from the first acquired frame, by this unitary emission rate to estimate the total number of TMC molecules within each fluorescence spot.

### Data availability

The data supporting the findings of this study are available from the corresponding authors upon request.

## Electronic supplementary material

Supplementary Information
