# Supplementary material for: Variable number of TMC1-dependent mechanotransducer channels underlie tonotopic conductance gradients in the cochlea
Source: Nat Commun. 2018 Jun 5;9:2185. doi: 10.1038/s41467-018-04589-8 (PMC5988745; doi:10.1038/s41467-018-04589-8)
Supplement: Supplementary file 1 — Supplementary Information [file 41467_2018_4589_MOESM1_ESM.pdf]

Supplementary Information

Variable number of TMC1-dependent mechanotransducer channels underlie tonotopic conductance gradients in the cochlea

Beurg et al., 2018

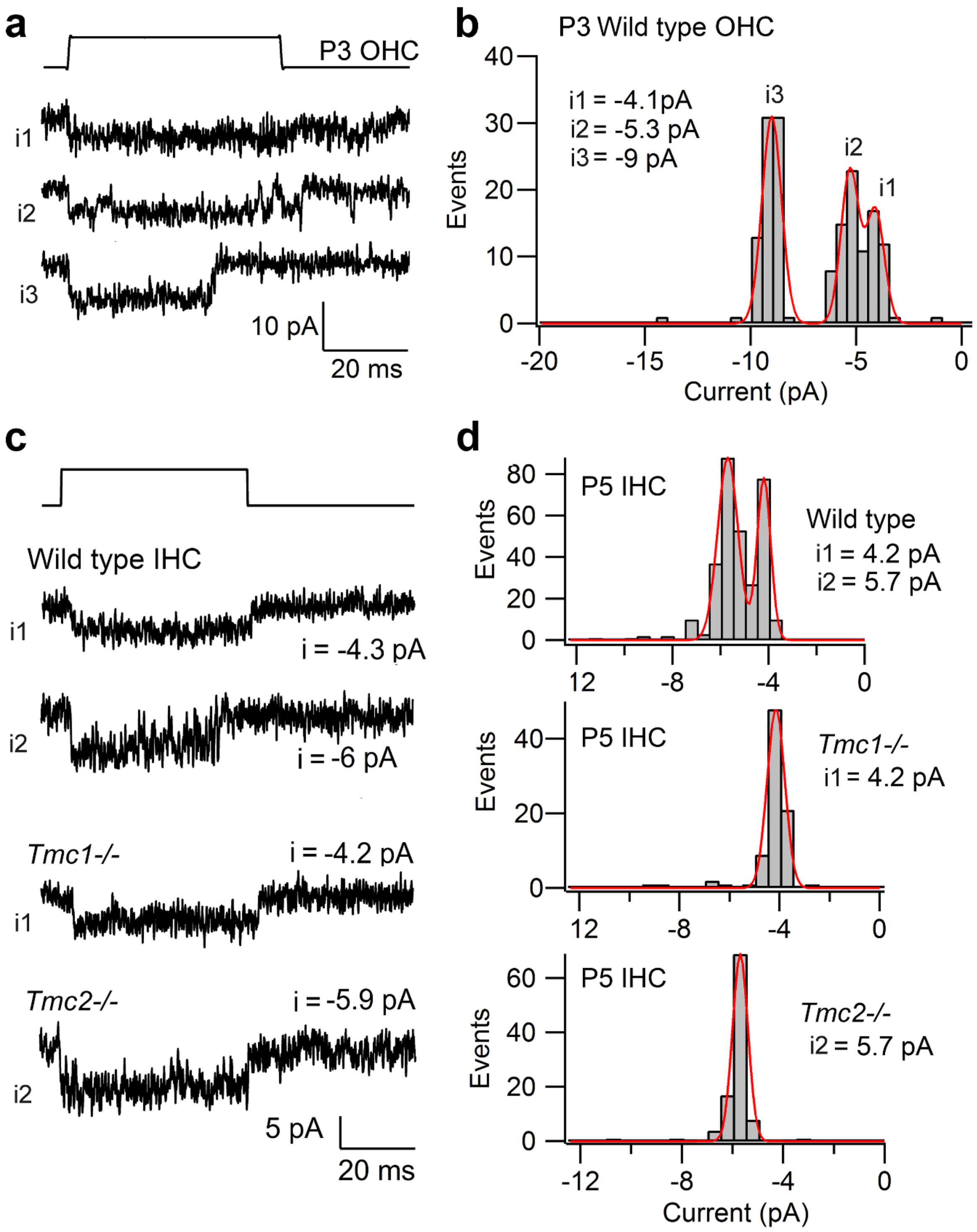

Supplementary Figure 1. Multiple single-channel current levels in wild-type hair cells.
a. P3 middle turn wild-type OHC recording, with three open channel current levels, i1, i2 and i3. **b** Histogram of open levels, i1 = 4.1 pA, i2 = 5.3 pA and i3 = 9 pA. These may correspond to TMC2 (i1) and TMC1 (i2, i3). **c.** Channel current levels in a wild-type IHC, in IHCs of *Tmc1*$^{-/-}$ and *Tmc2*$^{-/-}$ mice. All apical turn P5 IHCs. **d.** Histograms of current levels for three IHCs in **(c)**.

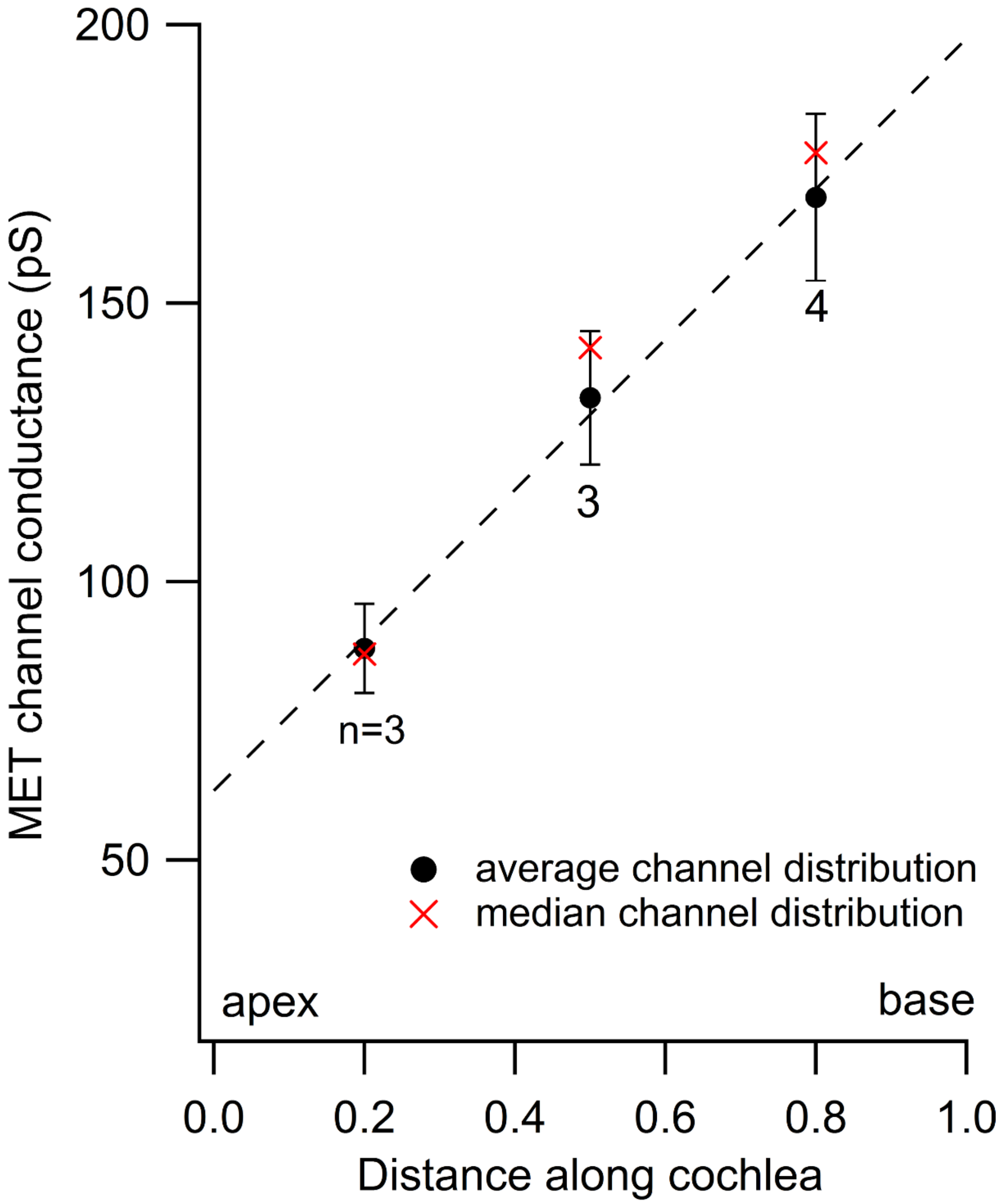

Supplementary Figure 2. Tonotopy of single-channel current levels in Tmc2$^{-/-}$ hair cells. MET channel conductances derived from mean of weighted averages of current distributions (filled circles, Mean ± S.D.; see text), and median channel conductances (red crosses) for apical turn, middle turn and basal turn OHCs. Number of cells is indicated on the graph.

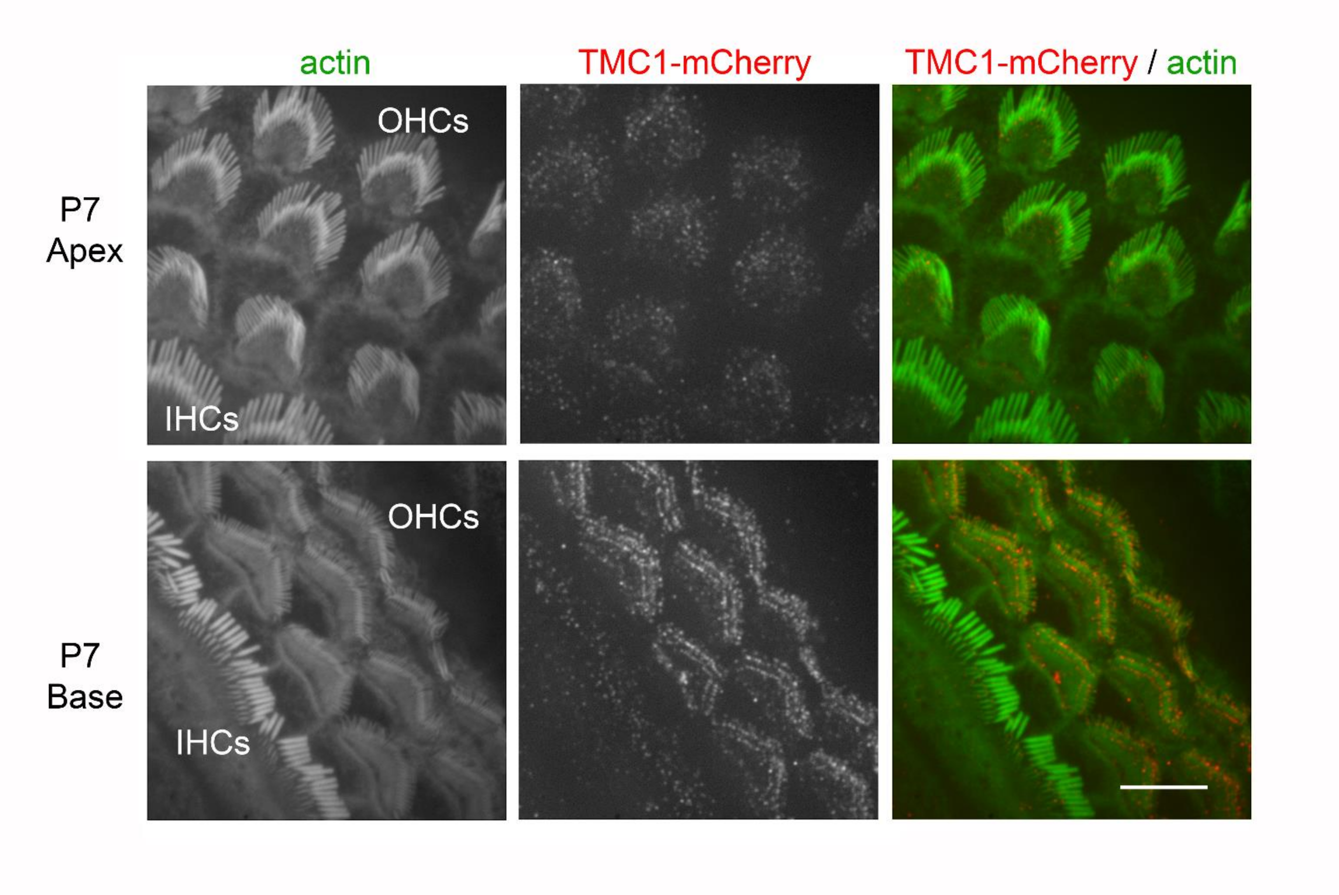

Supplementary Figure 3. TMC1-mCherry expression shows tonotopic gradient in OHC stereocilia at P7. Confocal images show OHC and IHC stereocilia bundles from transgenic mice expressing TMC1-mCherry. Fluorescence intensity of TMC1-mCherry puncta in OHC stereocilia increases from apex to base. Scale bar = 7µm.

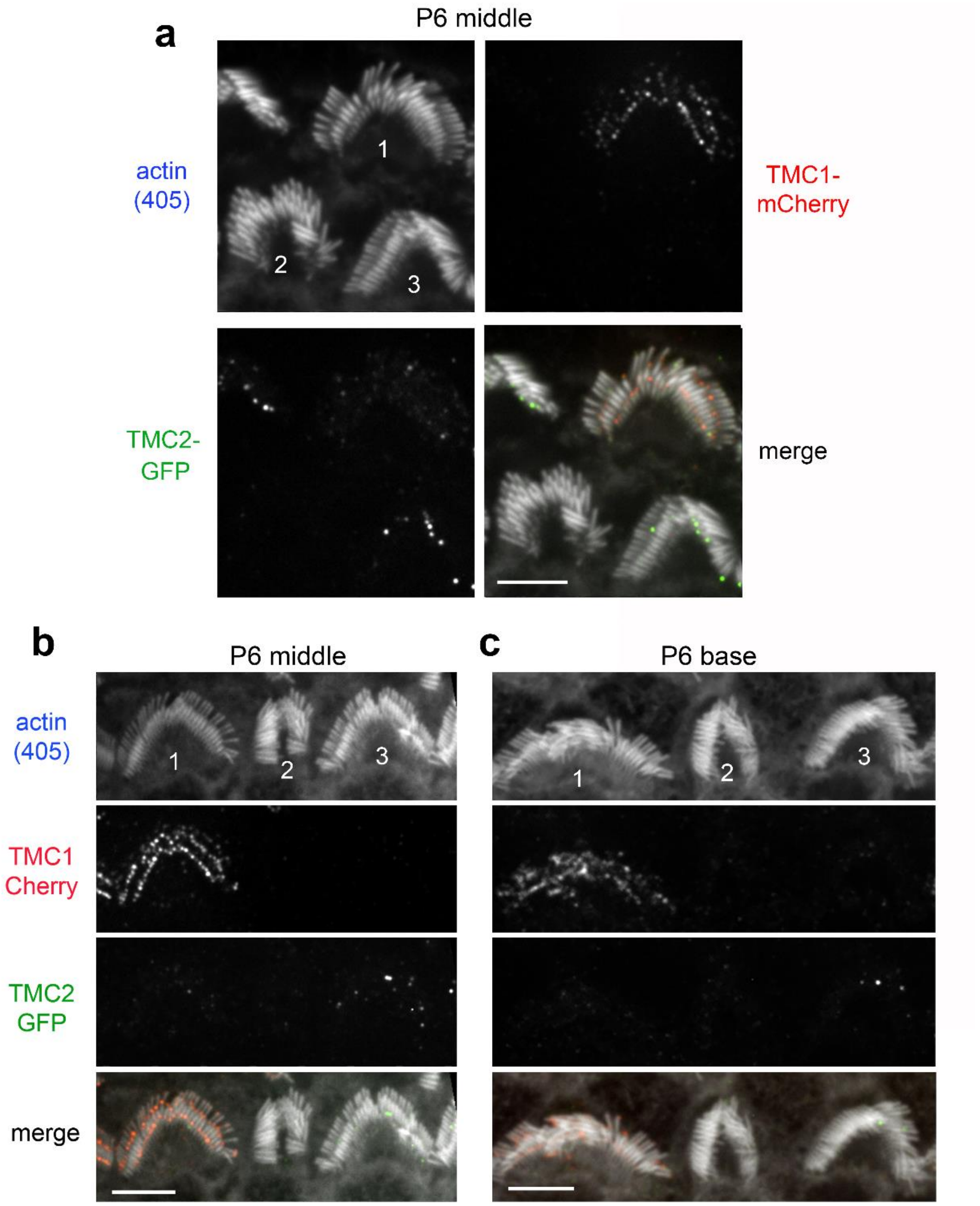

Supplementary Figure 4. Expression of TMC1 and TMC2 is necessary for normal OHC bundle morphology. a-c Confocal images of OHC stereocilia bundles from P6 mice with mosaic expression of TMC1-mCherry and TMC2-GFP at cochlear middle (**a** and **b**) and basal **(c)** turn. Lack of both TMC1-mCherry and TMC2-GFP (bundle 2 in **a-c**), results in abnormally small and rounded bundles. Presence of either TMC1-mCherry (bundle 1 in **a-c**) or TMC2-GFP (bundle 3 in **a** and **b**) is sufficient to maintain normal stereocilia staircase and bundle morphology in the cochlear middle turn. In the basal turn, TMC2-mCherry puncta are fewer in number and larger in size as expression is beginning to decline (bundle 3 in **c**). Here, TMC2-GFP alone is not sufficient to maintain stereocilia bundle morphology in absence of TMC1-mCherry. Scale bars = 5µm.
